# The Subcutaneous Implantable Cardioverter-Defibrillator: Not For All

**DOI:** 10.1016/s0972-6292(16)30772-0

**Published:** 2014-07-15

**Authors:** Krishnakumar Nair, Sheila Watkins, Douglas Cameron

**Affiliations:** Division of Cardiology, Department of Medicine, University of Toronto, Peter Munk Cardiac Centre, University Health Network, 200 Elizabeth Street, Toronto-M1R2G5

**Keywords:** Implantable Cardioverter-Defibrillator, Subcutaneous

## Introduction

Implantable cardioverter-defibrillators (ICDs) have a proven role in primary and secondary prevention of sudden death from ventricular arrhythmias. Transvenous ICD systems despite this proven benefit, have been plagued by lead problems including fracture, insulation breaks and recalls; and issues with transvenous access including pneumothorax and venous obstruction. Infected or fractured leads require full extraction which may require laser-assisted removal which carry a mortality and morbidity risk. Subcutaneous ICDs (S-ICD) are an exciting recently approved technology providing the advantages of effective shock rescue without need for transvenous leads. They function as essentially shock boxes without pacing capability except for a short period of post shock transcutaneous pacing. This technology provides an emerging option especially in patient groups who do not have a pacing indication, have no venous access, patients at high infection risk, patients with no structural heart disease and hereditary arrhythmias; and patients with congenital heart disease that precludes transvenous leads. Patients with slow monomorphic ventricular tachycardia consistently terminated by antitachycardia pacing are also not candidates for this therapy in evolution. In addition, S-ICDs do not have remote monitoring capability.

The S-ICD system uses modified subcutaneous electrocardiography through either a primary, secondary, or alternate vector, which detects changes in ventricular rate. The heart rate is measured as the rolling average of 4 consecutive sensed intervals. Ventricular fibrillation (VF) is diagnosed when 18 of 24 consecutive sensed events exceed the shock zone limit.

In this issue of the Indian Pacing and Electrophysiology Journal, Tekkatte et al [1] describe inappropriate therapies delivered from a subcutaneous ICD in their report titled "A Case of Inappropriate Therapies for Atrial Flutter From a Subcutaneous ICD". Inappropriate therapies were delivered three times in response to atrial flutter. One self-terminating episode of ventricular fibrillation was not detected. After failed ablation, more aggressive medical management of the atrial flutter was planned with Amiodarone and Bisoprolol. Despite programming changes in response, which included increasing the VF detection rate, and changing the vector for detection; the patient continued to get shocks from the device. Finally, a transvenous ICD was implanted.

Of note, the S-ICD device in this case was always programmed with one zone. Discriminator functions on the S-ICD are however possible only with dual zone programming into a shock zone and a conditional shock zone. There are no discriminators in the shock zone. Discrimination has 3 steps: 1- comparison of current tachycardia complex waveform with stored template acquired at rest (>50% = supraventricular tachycardia). 2- beat to beat analysis evaluates for polymorphic or monomorphic relationship (if polymorphic - ventricular tachycardia, if monomorphic - algorithm continues). 3 - QRS width analysis (wide - ventricular tachycardia, Narrow - supraventricular tachycardia).

There is early data now available regarding usefulness of these discriminators. In the START Study, Gold et al [2] showed 100% sensitivity and 98% specificity for VT and SVT with the S-ICD. Specificity was best with the S-ICD in a head-to-head comparison with transvenous discriminations algorithms from 3 different manufacturers. In another article by the same group (now in press in the Heart Rhythm journal) [3], 226 subjects (72%) with dual zone programming (which included a S-ICD discrimination algorithm) and 88 subjects (28%) with single zone programming were compared. The addition of a second shock zone with an active discrimination algorithm was strongly associated with a reduction in inappropriate shocks with the S-ICD system and did not result in prolongation of detection times or increased syncope. These data support the use of dual zone programming as a standard setting for S-ICD patients.

This discrimination algorithm however would not have helped in the reported case because it relies on comparing R wave to R wave. In the case study, flutter waves were oversensed as R waves and it is unlikely that they would match the template. The problem here was the far-field sensing vectors (8 cm shock coil separating sensing electrodes) and the large flutter waves.

Analogous to this case, in another patient with hypertrophic cardiomyopathy, we have seen flutter waves sensed as ventricular fibrillation signals in a transvenous ICD. The lead was repositioned which is not an option with the S-ICD. In addition, chances of cross chamber sensing are likely to be less due to bipolar near - field sensing in both atrium and ventricle with transvenous system. Pre- implant ECG assessment is primarily for T wave to R wave ratio and not atrial versus ventricular ratio and importantly the patient was not in flutter at time of assessment.

Anecdotal case reports now demonstrate compatibility with transvenous and epicardial pacemakers which will increase the clinical utility of S-ICD despite being limited in not having the capabilities of bradycardia pacing, anti-tachycardia pacing and cardiac resynchronization therapy. Superior discrimination algorithms in future iterations of this device may help expand implant indications further. However, there may remain situations like in the reported case where it may not be possible to tackle oversensing of large atrial electrograms by programming changes in a S-ICD in its current form.
